# Rearing pattern alters porcine myofiber type, fat deposition, associated microbial communities and functional capacity

**DOI:** 10.1186/s12866-019-1556-x

**Published:** 2019-08-06

**Authors:** Keke Qi, Xiaoming Men, Jie Wu, Ziwei Xu

**Affiliations:** Institute of Animal Science, Zhejiang Academy of Agricultural Sciences, 145 Shiqiao Road, Jianggan, Hangzhou, 310021 People’s Republic of China

**Keywords:** Rearing pattern, Myofiber type, Fat deposition, Gut micobiota

## Abstract

**Background:**

The Chinese believe that the meat of pigs reared in the past with free range tastes better than that of the pigs reared indoor on a large scale today. Gastrointestinal microflora is closely associated with the main factor of meat flavour, including fibre characteristics and lipid metabolism. Our method in this study involved different raising patterns within the semi free-grazing farm (FF) or indoor feeding farm (DF), the measurement of fat deposition and myofiber type by paraffin section and reverse transcription polymerase chain reaction and the identification of microbiome and functional capacities associated with meat quality through metagenomic sequencing.

**Results:**

Results showed that the fat area in muscle and adipose tissue and the myofiber density significantly increased in the pigs of the FF group. The relative abundance of bacteria associated with lipid metabolism, such as g_*Oscillibacter*, in the feces of the FF group was higher than that in DF group, and the relative abundance of some bacteria with probiotic function, including *g_Lactobacillus* and *g_Clostridium*, was lower than that in DF group. The abundance of *g_Clostridium* was significantly positively correlated with the intramuscular fat area, whereas health-related bacteria, such as *g_Butyricicoccus*, *g_Eubacterium*, *g_Phascolarctobacterium* and *g_Oribacterium*, was significantly negatively correlated with abdominal fat area, myofiber density and adipose triglyceride lipase (ATGL) mRNA expression. KEGG analysis showed that pigs raised in semi free-grazing farm can activate the pathway of inosine monophosphate (IMP) biosynthesis, glycolysis/gluconeogenesis and alanine, aspartate and glutamate metabolism.

**Conclusions:**

Free range feeding improves meat quality by changing the fibre type, myofiber density and metabolic pathways related to flavour amino acids, IMP or glycolysis/gluconeogenesis in muscle. However, prolonged feeding cycle increases fat deposition and associated microbial communities.

**Electronic supplementary material:**

The online version of this article (10.1186/s12866-019-1556-x) contains supplementary material, which is available to authorized users.

## Background

Except for the influences of nutrition, feeding management and slaughter mode, muscle fibre and intramuscular fat are the main factors affecting muscle quality, such as tenderness, juiciness and flavour. The number and volume of muscle fibres mainly determine the meat quantity, and the type of muscle fibre is closely related to the pH change, colour, and water-holding capacity of post-mortem muscles [1–3]. The content of intramuscular fat is closely related to tenderness, juiciness and flavour of the meat [4].

Intestinal flora and its metabolites are closely associated with the immune system, bone, cardiovascular system and nervous system and thus affect the physical and mental health [5, 6]. Most research on intestinal microorganism and lipid metabolism focus on metabolic disorders and obesity. Adult germ-free mice with microbiota harvested from the distal intestine of conventionally raised animals produces a 60% increase in body fat content and insulin resistance despite reduced food intake [7]. Gut microbiota is an important factor affecting energy harvest from the diet and energy storage of the host [8, 9]. Yan et al. (2016) demonstrated significant difference of the gut microbiota, which contributed in regulating fibre characteristics and lipid metabolism in skeletal muscle between obese and lean pigs [10]. Specific gut microbiome in obese individuals can enhance ectopic fat deposition in skeletal muscle and inhibit muscle growth [10]. These findings provide new approaches to intervene with host metabolism and animal phenotypes. Intestinal flora is an independent environmental factor for regulating host metabolism, and its changes are closely related to the host phenotype. However, the biological markers of intestinal microorganism related to phenotypic traits, such as muscle fibre and fat, have not been determined. A reference gene catalogue of pig gut microbiome has been established by Xiao et al. (2016) [11]. Their study would accelerate research about the complex interactions between intestinal microbiota and host physiological function.

Almost all the Chinese believe that pork raised in the past with free range tastes much better than pork raised indoor on a large scale today. On June 18, 2016, the famous international media New York Times published large-scale reports on Linan Sun Commune and China’s rural construction. They followed past pig raising model, where pigs were running in the yard, swimming in the pool, rolling in the mud, and using their mouths to arch the fruit tree fences. Hence, in this study, pigs in the same litter were raised separately. One part of the pigs was raised in traditional indoor farm, while the others were raised above Linan Sun Commune with the semi free-grazing model. The main factors affecting meat flavour, including fibre characteristics and lipid metabolism, and the correlation between porcine gut microbiome and muscle fibre or fat deposition would be tested, which will provide a theoretical basis for regulating pork quality through intestinal microflora and its metabolites.

## Results

### Measurement of fat deposition and myofiber type traits

**Fig. 1:**
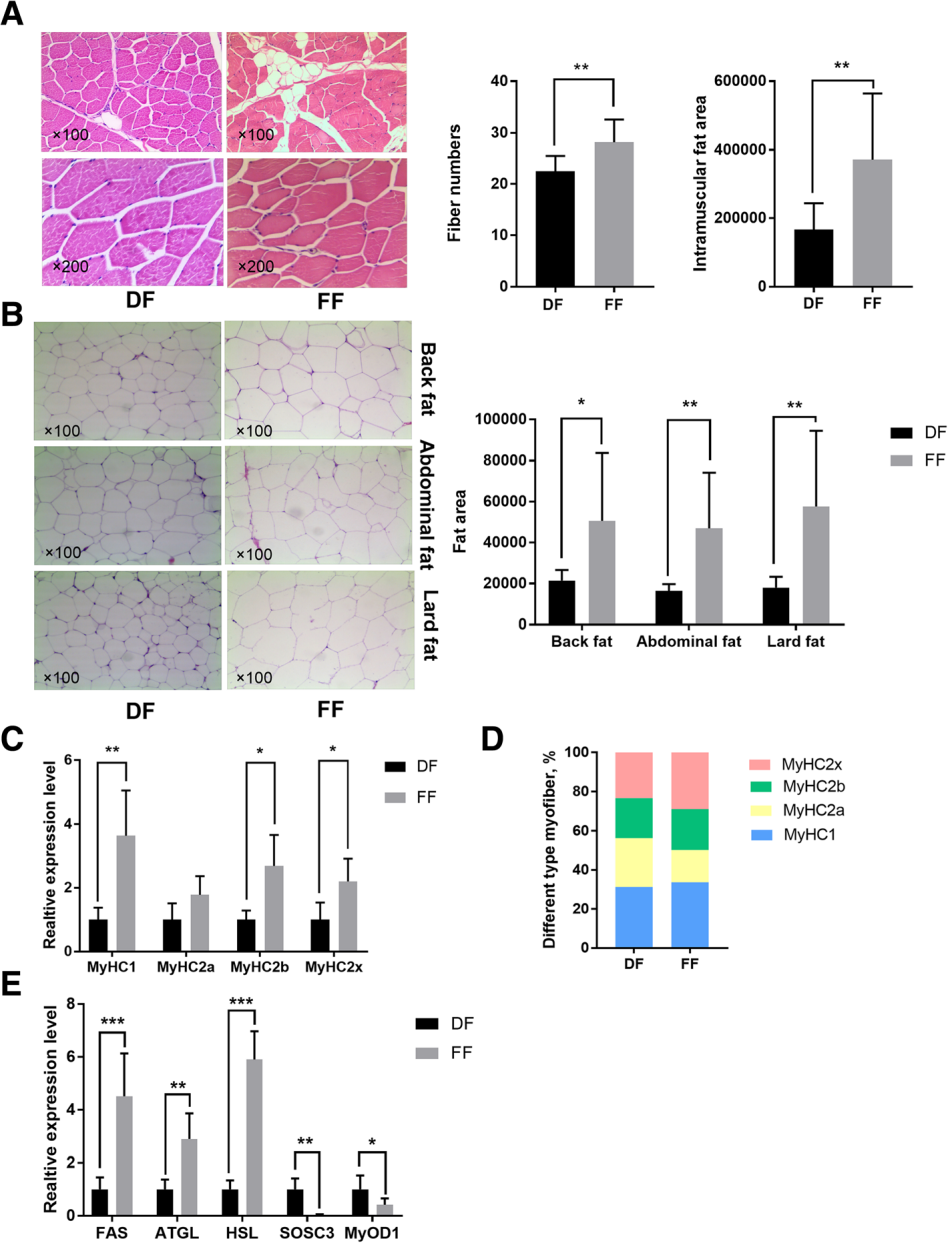
Fibre number and the intramuscular fat area of longissimus dorsi muscles in the DF and FF groups (**a**). Back, abdominal and lard fat area of pigs in the DF and FF groups (**b**). Gene expression of MyHC1, MyHC2a, MyHC2b and MyHC2x of pigs in the DF and FF groups (**c**). Myofiber type as indicated by the percentage of different MyHC in the longissimus dorsi muscles of pigs in the DF and FF groups (**d**). Gene expression of the fat deposition and meat quality of longissimus dorsi muscles in the DF and FF groups (**e**). * represents significant difference (*p* < 0.05), and ** represents significant difference (*p* < 0.01)

The intramuscular fat area and fibre number in the longissimus dorsi muscles of animals in the semi free-grazing farm (FF) group were significantly (*p* < 0.01) bigger than that in the indoor feeding farm (DF) group (Fig. 1a). Back, abdominal and lard fat area of pigs in the FF group were significantly (p < 0.01) bigger than that in the DF group (Fig. 1b). The mRNA expression of MyHC1, MyHC2b and MyHC2x in the longissimus dorsi muscles of animals in the FF group was significantly (*p* < 0.05) higher than that in the DF group (Fig. 1d). The percentage of MyHC2x and MyHC1 in FF group was higher than that in DF group (Fig. 1c). The mRNA expression of adipocyte development genes, including FAS, ATGL and HSL, in the longissimus dorsi muscles of animals in the FF group was significantly (*p* < 0.05) higher than that in the DF group (Fig. 1e). The mRNA expression of MyOD1 and SOSC3 in the longissimus dorsi muscles of animals in the FF group was significantly (p < 0.05) lower than that in the DF group (Fig. 1e).

### Taxonomic profiles in pig feces

Metagenome sequencing by using HiSeq Illumina platform produced 64,035.32 Mbp of raw data from 10 faecal samples (Additional file 4: Table S1). A total of 63,409.46 Mbp clean data were obtained after quality control. A total of 1,282,750,898 bp scaftigs were obtained, which were assembled from the single or mixed sample assembly. MetaGeneMark software predicted 1,992,770 open reading frames (ORFs). A total of 1,069,209 ORFs with 644.58 Mbp lengths, which include 348,172 complete genes, were obtained after redundanting. MicroNR database was used to blast with no-redundant gene catalogue, and 78.76, 75.37, 69.83, 69.35, 58.72, 55.00 and 41.31% at the kingdom, phylum, class, order, family, genus, or species level, respectively, were annotated using the lowest common ancestor (LCA) method. The observed numbers of non-redundant genes curves for all samples of FF and DF groups are shown in Additional file 1 :Figure S1.

**Fig. 2:**
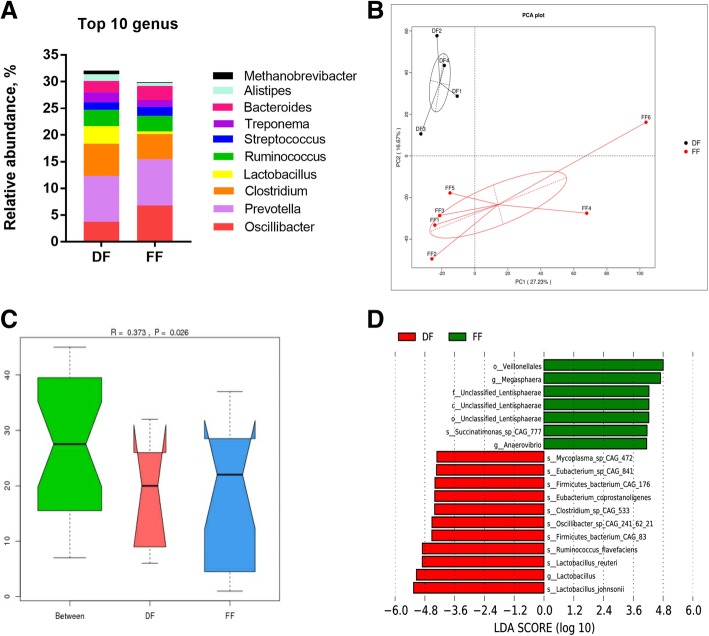
Relative abundances of the bacteria in the top 10 genus level taxa of the DF and FF groups (**a**). Principal component analysis generated using the species abundance based on the lined model dissimilarities. Each point represents a sample (**b**). Differences were assessed by ANOSIM, and significance was established at *p* < 0.05. *R* > 0 indicates that the difference between groups was greater than the difference within groups at the species level (**c**). Comparisons of faecal bacteria by using LefSe analysis. LDA scores indicates the significant diversity (p < 0.05) between the DF group and FF group at the species level (**d**)

A total of 1646 annotated genera were found in all samples. Differences were observed at the genus level between the DF and FF groups, indicating different microbial community profiles and abundances. The most dominant genera in the DF group were *Prevotella* (8.59%), *Clostridium* (6.05%), *Oscillibacter* (3.74%), *Lactobacillus* (3.28%), *Ruminococcus* (3.03%), *Bacteroides* (2.15%), *Treponema* (1.84%), *Streptococcus* (1.44%), *Alistipes* (1.24%) and *Methanobrevibacter* (0.68%), while those in the FF group included *Prevotella* (8.64%), *Oscillibacter* (6.81%), *Clostridium* (4.72%), *Ruminococcus* (2.90%), *Bacteroides* (2.65%), *Streptococcus* (1.73%), *Treponema* (1.25%), *Alistipes* (0.60%), *Lactobacillus* (0.48%) and *Methanobrevibacter* (0.10%) (Fig. 2a).

A total of 7016 species were annotated from the MicroNR database, accounting for an average of 9.71% or 11.40% of the total NR from the DF or FF group, respectively (Additional file 5: Table S2). *Firmicutes bacterium CAG:110* exhibited high abundances and was dominant in sample from DF (3.30%)) and FF (2.84%). The species were followed in abundances by *Lactobacillus johnsonii* (1.19%), *Prevotella sp. P5–92* (0.89%) and *Prevotella sp. P2–180* (0.74%) in the DF group and by *Ruminococcus sp. CAG:177* (1.65%), *Oscillibacter sp. PC13* (1.35%) and *Clostridium sp. CAG:138* (1.34%) in the FF group (Additional file 2 :Figure S2).

Principal component analysis (PCA) and ANOSIM distances were utilised to visualise the differences in taxa composition between groups. The PCA cluster showed an obvious separation between the DF and FF group (Fig. 2b), and the ANOSIM enhanced this dissimilarity (*R* = 0.373, *p* < 0.05) in the species level (Fig. 2c), wherein identical communities are given R statistic near 0, and completely distinct communities are given a value of + 1. LDA indicated that the bacteria were significantly reduced in the DF group compared with those in the FF group, which was composed of *s_Succinatimonas_sp_CAG_777* in species level and *g_Megasphaera* and *g_Anaerovibrio* in genus level (Fig. 4c). The bacteria were significantly increased in the DF group compared with those in the FF group, consisting of *s_Mycoplasma_sp_CAG_472*, *s_Eubacterium_sp_CAG_841*, *s_Firmicutes_bacterium_CAG_176*, *s_Eubacterium_coprostanoligenes, s_Clostridium_sp_CAG_533*, *s_Oscillibacter_sp_CAG_241_62_21*, *s_Firmicutes_bacterium_CAG_83*, *s_Ruminococcus_flavefaciens*, *s_Lactobacillus_reuteri and s_Lactobacillus_johnsonii* in the species level (Fig. 2d).

### Correlation between the phenotypic data and microbial abundance

**Fig. 3:**
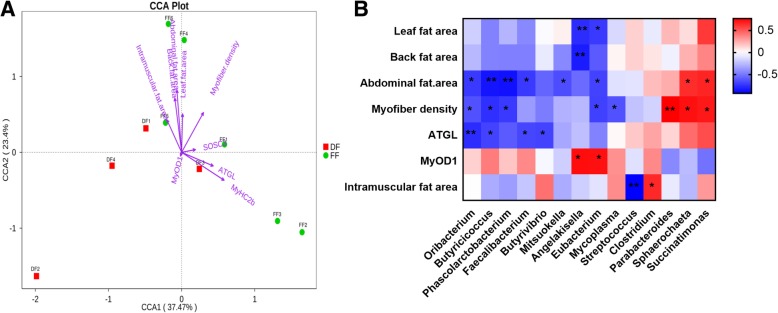
Multi canonical correlation analysis of the relative abundance of the genus in pig feces samples and environmental parameters. Environmental parameters include myofiber density, leaf fat area, abdominal fat area, back fat area, intramuscular fat area, MyOD1 mRNA and ATGL mRNA. Arrow lengths indicate the strength of the relationship between the environmental parameters and the overall microbial community (**a**). Heat maps of Spearman’s rank correlation coefficients between the myofiber density, leaf fat area, abdominal fat area, back fat area, intramuscular fat area, MyOD1 mRNA, ATGL mRNA and the relative abundance of bacterial genus (**b**). The correlation abundant coefficients are indicated by color, * represents significant correlation (p < 0.05), and ** represents significant correlation (*p* < 0.01)

We used the canonical correlation analysis (CCA) to detect the correlation between the eight selected environmental variables and the overall microbial community (Fig. 3a). CCA analysis showed that the abdominal fat area (*p* < 0.05), back fat area (*p* = 0.1) and MyHC2b (*p* = 0.1) had the greatest effect on microbial community composition, followed by myofiber density, leaf fat area, intramuscular fat area, ATGL mRNA, SOSC3 mRNA and MyOD1 mRNA. After applying CCA, all significant correlation between the selected phenotypic data and gut microbial by using the spearman rank correlation at the genus level are shown as a heat map in Fig. 3b. The effect size and direction of the correlation was presented by the fold change value and colour. In total, 27 significant correlations between individual variables and gut microbial were observed in multivariate analysis, including 8 positive correlations (red square) and 19 negative (blue square) correlations.

At the genus level, a significant positive correlation was observed in the intramuscular fat area of *g_Clostridium* (*R* = 0.65, *p* = 0.04), and a significant negative correlation was observed in the intramuscular fat area of *g_Streptococcus* (*R* = − 0.93, *p* = 0.0001) (Fig. 3b). A significant positive correlation was observed in myofiber density and the abdominal fat area of *g_Sphaerochaeta* (*R* = 0.72, *p* = 0.01; *R* = 0.64, *p* = 0.04) and *g_Succinatimonas* (*R* = 0.70, *p* = 0.02; *R* = 0.66, p = 0.04), whereas a significant negative correlation was observed in myofiber density and abdominal fat area of *g_Oribacterium* (*R* = − 0.64, *p* = 0.05; *R* = − 0.72, p = 0.02), *g_Phascolarctobacterium* (*R* = − 0.72, p = 0.02; R = − 0.87, *p* = 0.001)*, g_Eubacterium* (*R* = − 0.70, p = 0.02; *R* = − 0.68, *p* = 0.03) and *g_Butyricicoccus* (*R* = − 0.76, *p* = 0.01; *R* = − 0.85, p = 0.001)*.* A significant negative correlation was observed in ATGL mRNA expression of *g_Oribacterium* (*R* = − 0.77, *p* = 0.009), *g_Butyricicoccus* (*R* = − 0.68, *p* = 0.03), *g_Faecalibacterium* (*R* = − 0.67, *p* = 0.02) and *g_Butyrivibrio* (*R* = − 0.68, *p* = 0.03) (Fig. 3b). A significant positive correlation was observed in the myofiber density of *g_Parabacteroides* (*R* = 0.77, p = 0.009) (Fig. 3b)*.* A significant positive correlation in MyOD1 mRNA expression (*R* = 0.73, p = 0.01) and a significant negative correlation in leaf (*R* = − 0.77, p = 0.009) and back fat area (*R* = − 0.83, *p* = 0.003) were observed on *g_Angelakisella* (Fig. 3b). A significant positive correlation in MyOD1 mRNA (*R* = 0.72, p = 0.02) expression and a significant negative correlation in leaf (*R* = − 0.72, p = 0.02) and abdominal fat area (*R* = − 0.68, p = 0.03) were observed on *g_Eubacterium* (Fig. 3b).

### Functional composition of the metagenome analysis

**Fig. 4:**
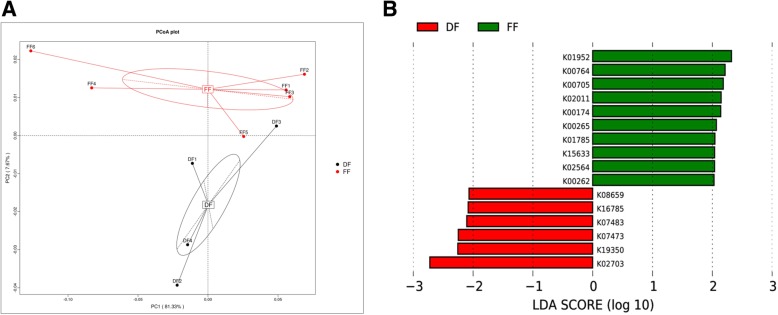
Two-dimensional principal component score plot based on the unweighted unifrac metrics for all samples. The KO (KEGG Orthology) detected in pig feces samples are shown for the DF group (black circles) and FF group (red circles) (**a**). Comparisons of KO level by using LefSe analysis. LDA scores indicates the significant diversity (p < 0.05) between DF group and FF group at the KO level (**b**)

Functional classification of annotated NR genes by using Kyoto Encyclopedia of Genes and Genomes (KEGG) revealed a predominance of pathways related to metabolism (carbohydrates, amino acid and nucleotide metabolism), genetic information processing (translation) and environmental information processing (membrane transport) as shown in Additional file 3 :Figure S3. PCoA clustered the two feeding models (DF vs. FF) distinctly with two PCoA axes describing 89% of the total variation, wherein 81.33% of variation was in the first principal axis (Fig. 4a). Chief differences between groups were reads that were functionally annotated as being involved in carbohydrate and amino acid metabolism. Interesting, samples from the FF group were significantly enriched for pathway to alanine, aspartate and glutamate metabolism by the action of glutamate dehydrogenase (K00262, EC:1.4.1.4), glutamate synthase and NADPH large chain (K00265, EC:1.4.1.13) and 2-oxoglutarate/2-oxoacid ferredoxin oxidoreductase subunit alpha (K00174, EC:1.2.7.3, 1.2.7.11). Moreover, the samples remarkably enriched pathway to glycolysis/gluconeogenesis by the action of aldose 1-epimerase (K01785, EC:5.1.3.3) and 2,3-bisphosphoglycerate-independent phosphoglycerate mutase (K15633, EC:5.4.2.12) and remarkably enriched the module to inosine monophosphate (IMP) biosynthesis by the action of phosphoribosylformylglycinamidine synthase (K01952, EC:6.3.5.3) and amidophosphoribosyltransferase (K00764, EC:2.4.2.14) (Fig. 4b).

## Discussion

### Raising pattern alter fibre density, MyHC I proportion and intramuscular fat area

The histological characteristics of muscle fibre are closely related to meat quality (flavour, juiciness and tenderness). Rearing conditions lead to substantial changes in the MyHC transformation as evidenced by the different proportions of myofiber types and differences in their myofiber numbers. Muscle tissue is composed of myofibers that can be subdivided into four interchangeable types, marked by the expression of four myosin heavy chain (MyHC) isotypes: one slow (MyHC I) and three fast MyHC isoforms (MyHC IIa, IIx and IIb) [12]. The shortening velocities increase in the following order: I < IIa < IIx < IIb [13] Muscle MyHC composition can be influenced by additional factors, such as animal nutrition, physical activity, age and environmental temperature [14]. Transition between MyHCs expressions follows reversible obligatory rules: I ⇄ IIa⇄IIx⇄IIb, and the type of muscle fibres changes to left or right with the external conditions [15].

In comparison with the DF group, percentage changes of MyHCs in the FF group were only found between oxidative myofibers: I ← IIa → IIx, whereas no changes were observed in the percentage of MyHC IIb. The main reason for the increased percentage of slow-twitch type I myofibers in the FF group in our study may be explained by the different raising condition. As a comprehensive factor, raising pattern had a substantial influence on muscle fibre composition. In our study, pigs in the DF group were kept under indoor breeding conditions and fed with a conventional commercial diet ad libitum, while the pigs in FF group were raised in a large yard and were highly physically active daily. This result was consistent with the report of Fazarinc et al. (2017) [16], wherein the percentage of MyHC-I was higher in wild pigs breeding outside, which were physically active than domestic pigs indoors. Higher percentage of slow-twitch type I myofibers was also found in wild pigs kept in group housing with diminished activity and was fed ad libitum [17] or was obtained from a zoological garden [18]. MyHC differentiation was accompanied by the myofiber density. Fazarinc et al., (2017) [16] found myofiber hypertrophy in domestic pig of all myofiber types, which was especially more intense than in the wild pig feeding outside. The same results were found in our study, wherein the fibre number in the longissimus dorsi muscles of animals in the FF group was significantly higher than that in the DF group.

The composition of skeletal muscle fibre type was related to meat quality, lipid content and distribution in muscle, which directly affects muscle quality [19]. The small muscle fibre area and high density are consistent with the prominent meat quality. The diameter of type I muscle fibres is small, and the number of muscle fibres per unit area is large. Hence, the intramuscular fat (IMF) content is high, and IMF affects the tenderness and flavor of muscle. The expression of MyHC I and IIa was positively correlated with IMF, whereas the expression of MyHC IIb was negatively correlated with IMF [20]. Hence, an increase in IMF can enhance the eating quality of pig meat [21], and MyHC I myofibers and fibre density are beneficial to pork quality [22].

Intramuscular accumulation of lipids and myofiber type of skeletal muscles is the result of interactions between genetical background and environmental factors. Other explanation for increasing IMF and levels of type I fiber in longissimus dorsi muscles of animals in FF group maybe influence by some bioactive compounds in diet, the longer feeding cycle. For example, the bioactive compounds in mulberry leaves may play a regulatory role in fat deposition and muscle fiber types, like resveratrol, anthocyanin or 1-deoxynojirimycin [23]. A decreased expression of fast and an increased expression of slow MyHC occurred in many physiological situations including ageing was demonstrated in studies in variety of species [24]. Prolonged feeding cycle of pigs in the FF group increased fat deposition, as indicated by increasing back, abdominal, lard and intramuscular fat area in the longissimus dorsi muscles. In brief, pigs reared in semi free-grazing farm increased fibre density, the MyHC I proportion and intramuscular fat area in this study, which could partly explain the better taste of free range pork.

### Raising pattern alter intestinal microbial

The effect of feeding pattern on intestinal microorganisms was studied. Prolonged of feeding cycle of pigs in the FF group increased fat deposition. The ratio of *Firmicutes*/*Bacteroidetes* (F/B) was associated with the energy harvest and fat deposition of host [8], and the F/B ratio seemed as a biomarker indicative to obesity. However, with the rapid accumulation of data by meta-analyses, a clear trend between the F/B ratio and obesity status could not be found [25], which suggested that the complexity of how gut microbiome modulates obesity is way more than a simple imbalance in the status of these phyla. The abundance of *Oscillibacter* was the major determinant of obese or normal status [26], when *Bacteroides* and *Faecalibacterium* were equally abundant. As a conditional pathogenic bacterium, *Oscillibacter* was positively associated with obesity-related metabolic pathways [27]. Consistent with our study, *g_Oscillibacter* in feces was relative higher in higher fat deposition group. In the top 10 genera, the relative abundance of *g_Lactobacillus* was relative higher in the DF group. Linear discriminant analysis effect size (LEfSe) analysis found that *s_Lactobacillus_johnsonii* and *s_Lactobacillus_reuteri* were significantly higher bacterial species in the DF group. *Lactobacillus* is recognised probiotics in animal husbandry, which compete for nutrients with existing gut microbiota, which reduce body weight and fat mass [28]. The relationship of *g_Lactobacillus* and obesity are controversial. Among the bacterial genera, *Lactobacillus* increased after a weight loss program in adolescents [29], but it was also abounded in obese and overweight children [30]. Million et al. (2012) [31] demonstrated that *s_Lactobacillus_reuteri* was associated with obesity in adults. These findings suggest a possible role of *Lactobacillus* at the species level in body weight and obesity. From the LEfSe analysis at the species level, significantly higher bacterial species was found in the FF group *s_Firmicutes_bacterium_CAG_176*, *s_Oscillibacter_sp_CAG_241_62_21* and *s_Firmicutes_bacterium_CAG_83*, which all belong to *Clostridium* cluster IV. *Clostridium* cluster IV is a dominant bacterial group known as butyrate-producing bacteria [32], which have beneficial functions, including nutrient absorption, epithelial cell maturation, production of short chain fatty acids and maintenance in human [33]. The abundance and diversity of *Clostridium* cluster IV decreases in vegetarians [34]. *s_Ruminococcus_flavefaciens* was another higher bacterial species in the DF group. Ransom-Jones et al. (2012) [35] found that *R. flavefaciens* and *Clostridium* cluster IV degraded dietary fibre. Above all, bacteria related probiotic function, including *g_Lactobacillus* and *Clostridium* cluster IV abound in DF group, and the relative abundance of *g_Oscillibacter*, which was associated with obesity, was higher in the FF group.

### Relationship between faecal microbial and myofiber types or fat deposition

Reports about the relationship between faecal microbial and muscle fibre types or fat deposition were rare. Yan et al. (2016) demonstrated abundance levels of major bacteria that produce butyrate and acetic acid, such as species from *Roseburia* and *Blautia*, were higher in obese Rongchang pigs and its mouse recipients [10]. In our study, bacterial genera *g_Butyricicoccus, g__Eubacterium, g_Phascolarctobacterium* and *g_Oribacterium* had significant negative correlation with abdominal fat area, myofiber density and ATGL mRNA expression. *Butyricicoccus* is a butyrate-producing clostridial cluster IV genus. The abundance of *Butyricicoccu*s increased significantly with age [36]. Genera *Eubacterium* of the gut microbiota belongs to Clostridium clusters XIVa, and are known for polysaccharide fermentation and bile acids dehydroxylation [37]. Patients with poorer nutritional status revealed a much lower abundance of Clostridium cluster XIVa compared to healthy siblings with poorer nutritional status [38]. Many Clostridium clusters IV and XIVa bacteria produce butyrate, a metabolite that acts as a primary source of energy for colon cells, helps eliciting an anti-inflammatory response, establishment and maintenance of the GI barrier, and reduction of intestinal permeability [39, 40]. Clostridial cluster XIVa bacteria have potential beneficial effects with respect to the development of obesity and associated metabolic disorders [41]. Clostridium cluster IV, which has been associated with both obesity and weight loss [29, 42]. The higher levels of Clostridium cluster IV were detected in the Swedish with a high consumption of fish and meat [43], and lower abundance of Clostridium cluster IV in faecal microbiota of vegetarians [34]. *g*_*Oribacterium* is a health-associated genus [44], which decreased in chronic obstructive pulmonary disease patients. *Phascolarctobacterium* may exert a beneficial role in the human gastrointestinal tract and can produce short-chain fatty acids, including acetate and propionate [45, 46]. Besides, *Phascolarctobacterium spp*. specialise in the utilisation of succinate produced by other bacteria. The abundance of *Parabacteroides*, which is a major producer of succinate, was increased by high fat diet and was positively correlated with body weight [47]. *Phascolarctobacterium faecium*-like bacteria in younger individuals were maintained at a high level with a gradual increase with increasing ages (between 1 and 60 years old) but with a decrease in elderly individuals (> 60 years old) [45]. In brief, health related bacteria had negative correlation with abdominal fat area, myofiber density and ATGL mRNA expression. The report about relationships between relevant microbiota and phenotypes of meat quality is rare, further research is needed.

### Semi free-grazing feeding activated metabolic pathway related with meat flavour

Sixteen functional KO were detected to be differentially abundant in at least one of the two raising patterns. Ten of these KO were significantly enriched in FF group compared with the DF group. Interestingly, two metabolic KO (K01952 and K00764) were involved in the modules of IMP biosynthesis (M00048), which is the pathway module for phosphoribosyl pyrophosphate + glutamine = > IMP [48]. Additionally, five KO detected in FF group were responsible for alanine (Ala), aspartate (Asp) and glutamate (Glu) metabolism (K00262, K00265 and K00174) and glycolysis/gluconeogenesis (K01785 and K15633). ABC transport pathway (K16785 and K19350), deoxyribonucleic acid (DNA)-damage-inducible protein (K07473) and transposase (K07483) were significantly overrepresented in the DF group. They are all associated with DNA repair processes.

Glu, Asp and Ala are important flavour amino acids, which can act on the precursor amino acid as a fundamental substance forming delicate flavour of meat; especially, Glu is the most pivotal amino acid affecting meat flavour and acid-base buffering capacity [49, 50]. By the KEGG pathway analysis, the *Clostridium butyricum* -treated group enriched alanine, aspartate and glutamate metabolism provided supporting evidence for the increased meat quality in breast muscle of ducks [51, 52]. Significant alterations were observed in the alanine, aspartate and glutamate metabolism pathway with the treatment ofβ-Methylamino-l-alanine [53]. IMP is the key indicator for meat flavor [54–56]. IMP and inosine degrade into active ribose and ribose phosphate, which participate in the Maillard reaction in meat during cooking and produce various volatile flavour compositions [57]. IMP and sodium glutamate can co-participate in the regulation of meat flavour [58]. The genes encoding the modules of IMP biosynthesis (M00048) in the cells of Se-enriched *C. utilis* were up-regulated according to KEGG pathway modules analysis [59]. More sequences related to inosine monophosphate (required for the de novo synthesis of purines) were identified from *O. flexuosa* transcripts than from the nearly-complete genome of *B.malayi* [60]. Glycolysis/gluconeogenesis is also a muscle-development-related pathway, which is strongly connected and belongs to the most important energetic processes that influence the muscle-to-meat conversion [61]. Drip loss strongly depends on post mortem energetic processes in the muscle. Julia et al. (2016) [62] found that glycolysis/gluconeogenesis significantly influences drip loss. Glycolytic potential can also predict meat quality, which is related with high drip loss and low pH_24h_ [63]. A report aimed to examine the specific genetic contribution of each breed to meat quality found that most of the differentially expressed genes were grouped in the Glycolysis/Gluconeogenesis pathway, which was over-expressed in Maremmana and down-expressed in Chianina [64]. They hypothesized that the high expression levels of genes involved in Gluconeogenesis due to that the Maremmana is a breed, which adapted to the harsh environment o and to poor forage. The glycolytic pathway was enhanced in oysters with high-glycogen content, and these oysters have a high-energy metabolism, as well as an increased antioxidant capacity and stress resistance [65]. Notably, the enrichment of KO involved in flavour amino acid metabolism, IMP biosynthesis and glycolysis/gluconeogenesis pathway in the longissimus dorsi muscle provides supporting evidence for the increased porcine meat quality in the FF group. However, more metagenome samples are needed for accurately statistical validation of these characterised KO as promising biomarkers for meat quality.

## Conclusions

In conclusion, pigs reared in semi free-grazing farm increased myofiber density, MyHC I proportion and intramuscular fat area. It also activated the metabolic pathways related to meat flavour, including flavour amino acid metabolism, IMP biosynthesis and glycolysis/gluconeogenesis. These findings provide evidence for the good taste of free range pork. Prolonged feeding cycle of pigs in the FF group increased the conditional pathogenic bacteria associated with obesity.

## Methods

### Animal sample collection

Animal studies were conducted in accordance with the guidelines of the Zhejiang Farm Animal Welfare Council of China and approved by the ethics committee of Zhejiang Academy of Agricultural Sciences. Twenty Bajiazhe black piglets from Jicheng Animal Husbandry Co., Ltd. were divided into two groups of 10 at 3 months of age with equal representation of littermates and sex. Piglets in the group of the indoor feeding farm (DF) was fed with general compound feed (Additional file 6 :Table S3) as recommended by National Research Council (NRC, 1998) and were housed in Jicheng Animal Husbandry Co., Ltd. Piglets in the semi free-grazing farm (FF) group were fed with compound feed with seasonal pastures and vegetables (Additional file 6 :Table S4) and were housed in Hangzhou Sun Commune. Faecal samples in the DF group and FF group were collected from each animal at 7 and 10 months of age respectively. Feces was collected rectally in each animal by using a sterile cotton swab wet, and then the tube with feces were rapidly frozen in liquid nitrogen and stored at − 80 °C until DNA extraction. Animals in the DF group or the FF group were slaughtered in the fixed-point slaughter house according the operating procedures of pig slaughtering (GB/T 17236–2008) with approximately 100 kg body weight at 7 or 10 months of age, respectively. Approximately 1 m^3^ back fat or abdominal fat was collected from the junction of the sixth and seventh thoracic vertebra. Approximately 1 m^3^ leaf fat was collected from perirenal fat. Exactly 5-cm-thick longissimus dorsi muscle from the last third or fourth rib backwards was collected from the carcass within 30 min after exsanguination. One piece of longissimus dorsi muscle was placed in the RNA locker immediately for RNA isolation. The other piece of longissimus dorsi muscles, back fat, abdominal fat and leaf fat were fixed in 10% formalin for histology.

### Paraffin section

The muscle and fat samples were placed into 10% formaldehyde (pH 7.4) for 12–24 h and then dehydrated and subjected to routine haematoxylin eosin staining. The muscle tissue was randomly selected at 5–10 visual fields at 100× (intramuscular fat area measurement) or 200× magnification (muscle fibre number and single muscle fibre area measurement) visual field. Then, the intramuscular fat area and single muscle fibre area were measured using Image-ProPlus 5 image analysis software. The adipose tissue was randomly selected at five visual fields at a 50× magnification visual field. Then, the Image-ProPlus 5 image analysis software was used to measure the adipocyte diameter of each sample (15 cells per specimen), as well as the area of the single fat.

### Reverse transcription polymerase chain reaction (RT-PCR)

Total RNA was extracted according to the RNAiso Plus instructions. According to the PrimeScript® RT-reagent Kit manual, the total RNA was used for first strand cDNA synthesis. The primers used are shown in Table S5. Real-time PCR was carried out with ABI plus one (Life Technologies, Carlsbad, CA, USA) by using SYBR Premix Ex TaqTM. Two-step PCR amplification was performed under the following conditions: 95 °C for 30 s, 40 cycles of 95 °C for 5 s and 62 °C for 34 s. Melting curve analysis was performed to verify the single-product generation at the end of the assay. Standard curves were generated based on the data obtained from the standards of the 2~2^− 6^ dilution series template. The amplification efficiency ranged from 90%~ 110%. Therefore, the relative level of gene expression between different groups can be calculated using the 2^-△△Ct^ method.

### DNA extraction

Total genomic DNA from was extracted using the hexadecyltrimethylammonium bromide method. The concentration of the extracted DNA was measured using 1% agarose gel electrophoresis. DNA purity was checked using Nano Photometer® spectrophotometer (IMPLEN, CA, USA). OD260/OD280 ≈ 1.8 was a qualified DNA sample. Then, the sample was diluted to 1 ng/μL by using sterile water.

### DNA library construction and sequencing

Exactly 1 μg DNA per sample was used as input material for the DNA sample preparations. Sequencing libraries were generated using NEBNext® UltraTM DNA Library Prep Kit for Illumina (NEB, USA) following the manufacturer’s recommendations, and index codes were added to attribute sequences to each sample. Briefly, the DNA sample was fragmented by sonication to a size of 350 bp. Then, DNA fragments were end-polished, A-tailed and ligated with the full-length adaptor for Illumina sequencing with further PCR amplification. At last, PCR products were purified (AMPure XP system), and libraries were analysed for size distribution in an Agilent2100 Bioanalyzer and quantified using real-time PCR. Index-coded samples were clustered on a cBot Cluster Generation System according to the manufacturer’s instructions. After cluster generation, the library preparations were sequenced on an Illumina HiSeq platform, and paired-end reads were generated.

### Quality control of reads

Clean data were obtained from raw data through quality control by using Readfq (V8, https://github.com/cjfields/readfq). To prevent host DNA contamination, it needs to be compared with the host database to filter out the reads that may originate from the host by using SoapAligner software (soap2.21, https://omictools.com/soapaligner-tool) with parameters [66] ‘identity ≥90%, -l 30, -v 7, -M 4,-m 200,-x 400’.

De novo assembly of the short reads. Clean data were assembled using the SOAPdenovo software [67] (V2.04, https://omictools.com/soapdenovo-tool) with parameters [68] ‘-d 1, -M 3, -R, -u, -F, -K 55’. Scaftigs without N were obtained by interrupting assembled scaffolds from the joint of N. Unused reads were obtained by mapping clean data against scaftigs by using SoapAligner software (soap2.21) with parameters ‘identity ≥90%, −m 200 -× 400’ [53]. Then, unused data were pooled using SOAPdenovo software (V2.04) with the same parameters of single sample [69]. Mixed assembled scaffolds were split into scaftigs, and scaftigs of lengths less than 500 bp were discarded.

### Gene prediction and gene abundance profiling

MetaGeneMark(V2.10, http://topaz.gatech.edu/GeneMark/) was used to predict ORFs in scaftigs from the single or mixed sample (> = 500 bp) [69]. Information was screened using a 100 nt cut-off with default parameters. Non-redundant initial gene catalogue was obtained using the CD-HIT software (V4.5.8, http://www.bioinformatics.org/cd-hit/) from predicted ORFs with parameters ‘-c 0.95, -G 0, -aS 0.9, -g 1, -d 0’ [70]. SoapAligner (soap2.21) was used to align paired-end clean data against the initial gene catalogue with parameters ‘-m 200, −× 400, identity ≥95’ [68]. Unigenes were obtained from paired-end reads by removing genes with reads ≤2. We counted the gene abundance according to both numbers of paired-end reads and gene length, which was calculated as follows:

$$ {G}_k=\frac{r_k}{L_k}\cdot \frac{1}{\sum \limits_{i=1}^n\frac{r_i}{L_i}} $$

Core-pan gene analysis, correlation analysis and Venn diagram of gene numbers were employed based on the gene abundance in each sample.

### Population stratification

Bacteria, fungi, archaea and viruses in the non-redundant (NR) library (Version: 20161115, https://www.ncbi.nlm.nih.gov/) of NCBI were used as reference database, and DIAMOND software (V0.7.9, https://github.com/bbuchfink/diamond/) [71] was used in sequence blast with parameters ‘blastp,-e 1e-5’. Results of evalue > minimum evalue*10 were removed [72]. To avoid multiple alignment results, we used the LCA (https://en.wikipedia.org/wiki/Lowest_common_ancestor) was used to identify species annotation of the sequence. Then, the species abundance and gene numbers of the six-level taxonomic classification from phylum to species of each sample were obtained. Then, Krnoa analysis [73], relative abundance profile, heat map of abundance clustering, PCA (R ade4 package, Version 2.15.3) [74] and NMDS (R vegan package, Version 2.15.3) [75]. Dimension reduction analysis was conducted based on the abundance of each classification. Difference between groups were found using ANOSIM analysis (R vegan package, Version 2.15.3). Differential species were found using Metastat and LEfSe statistical analysis. The *p* value of Metastat statistical analysis was obtained using permutation test of each classification between groups; then, the q value was obtained by the correcting p value by using the Benjamini and Hochberg false discovery rate method [76]. LEfSe software was used for LEfSe statistical analysis with the LDA score 2 [77].

### Gene functional classification

DIAMOND software (V0.7.9) was used to blast unigenes with function databases with parameters ‘blastp, -e 1e-5’. The function databases included the KEGG database (Version 201,609, http://www.kegg.jp/kegg/). Best blast hit (one HSP > 60 bits) from above blast was selected for subsequent analysis. The relative abundance of different function levels (five levels in KEGG database) was counted. Based on the abundance of different function levels, the numbers of annotated gene, relative abundance profile and heat map of abundance clustering were obtained, and PCA and NMDS dimension reduction analysis, ANOSIM analysis, metabolic pathway analysis and Metastat and LEfSe statistical analysis were carried out.

Correlation between the phenotypic data and microbial abundance. The correlation and p value between the phenotypic data and microbial alpha diversity or species were obtained using the Spearman correlation model. Windrose figures were constructed using the correlation data between phenotypic data and the top 35 genus or species abundance. Count value in figure was correlation coefficient ‘*r*’. *r* < 0 indicates significant negative correlation, whereas *r* > 0 indicates significant positive correlation. The absolute value of ‘*r*’ was found in the windrose figure. *p* < 0.05 was considered statistically significant.

### Statistical analysis

Data of the paraffin section and real-time PCR were expressed as mean ± standard deviation. Statistical differences between groups were determined by Analysis of Variance with Duncan’s multiple comparison. p < 0.05 was considered statistically significant.

## Additional files

Additional file 1: **Figure S1.** Observed number of non-redundant genes of the 10 total samples. (TIFF 168 kb)

Additional file 2: **Figure S2.** Relative abundances of the bacteria in the top 10 species level taxa of the DF and FF groups. (TIF 275 kb)

Additional file 3: **Figure S3.** Functional classification of annotated NR genes by using KEGG analysis. (TIF 304 kb)

Additional file 4: **Table S1.** Total statistic information of the metagenome sequencing by using HiSeq Illumina platform from 10 faecal samples. (XLS 57 kb)

Additional file 5: **Table S2**. Absolute abundance of unigenes in species level. (XLS 1105 kb)

Additional file 6: **Table S3**. Composition of the basal diets in the traditional feeding farm. **Table S4**. Composition of the basal diets the semi free-grazing farm. **Table S5**. Primer sequences (5′to 3′) used for the quantitative polymerase chain reaction. (DOC 59 kb)

## Data Availability

The datasets used and/or analysed during the current study are available from the corresponding author on reasonable request. All data generated or analysed during this study are included in this published article and its supplementary information files.

## Abbreviations

Ala: alanine
Asp: aspartate
CCA: canonical correlation analysis
DF: indoor feeding farm
DNA: deoxyribonucleic acid
F/B: Firmicutes/Bacteroidetes
FF: semi free-grazing farm
Glu: glutamate
IMF: intramuscular fat
IMP: inosine monophosphate
KEGG: Kyoto Encyclopedia of Genes and Genomes
KO: KEGG orthology
LCA: lowest common ancestor
LEfSe: linear discriminant analysis effect size
MyHC: myosin heavy chain
NR: non-redundant
ORFs: open reading frames
PCA: principal component analysis
PCR: reverse transcription polymerase chain reaction
