# Supplementary figures and images for: Rearing pattern alters porcine myofiber type, fat deposition, associated microbial communities and functional capacity
Source: BMC Microbiol. 2019 Aug 6;19:181. doi: 10.1186/s12866-019-1556-x (PMC6683424; doi:10.1186/s12866-019-1556-x)

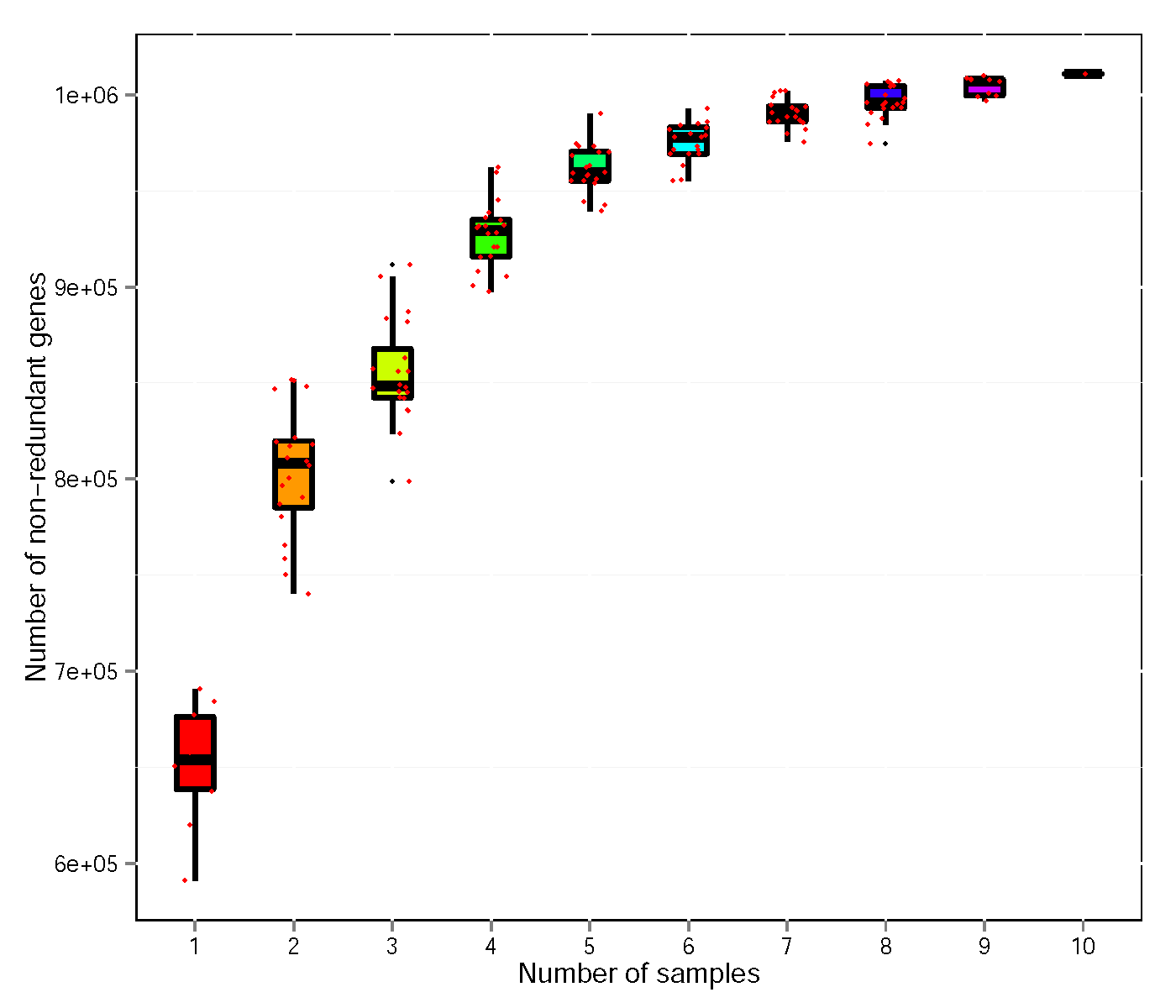

Supplement: Supplementary file 1 — Figure S1. Observed number of non-redundant genes of the 10 total samples. (TIFF 168 kb) [file 12866_2019_1556_MOESM1_ESM.tiff]

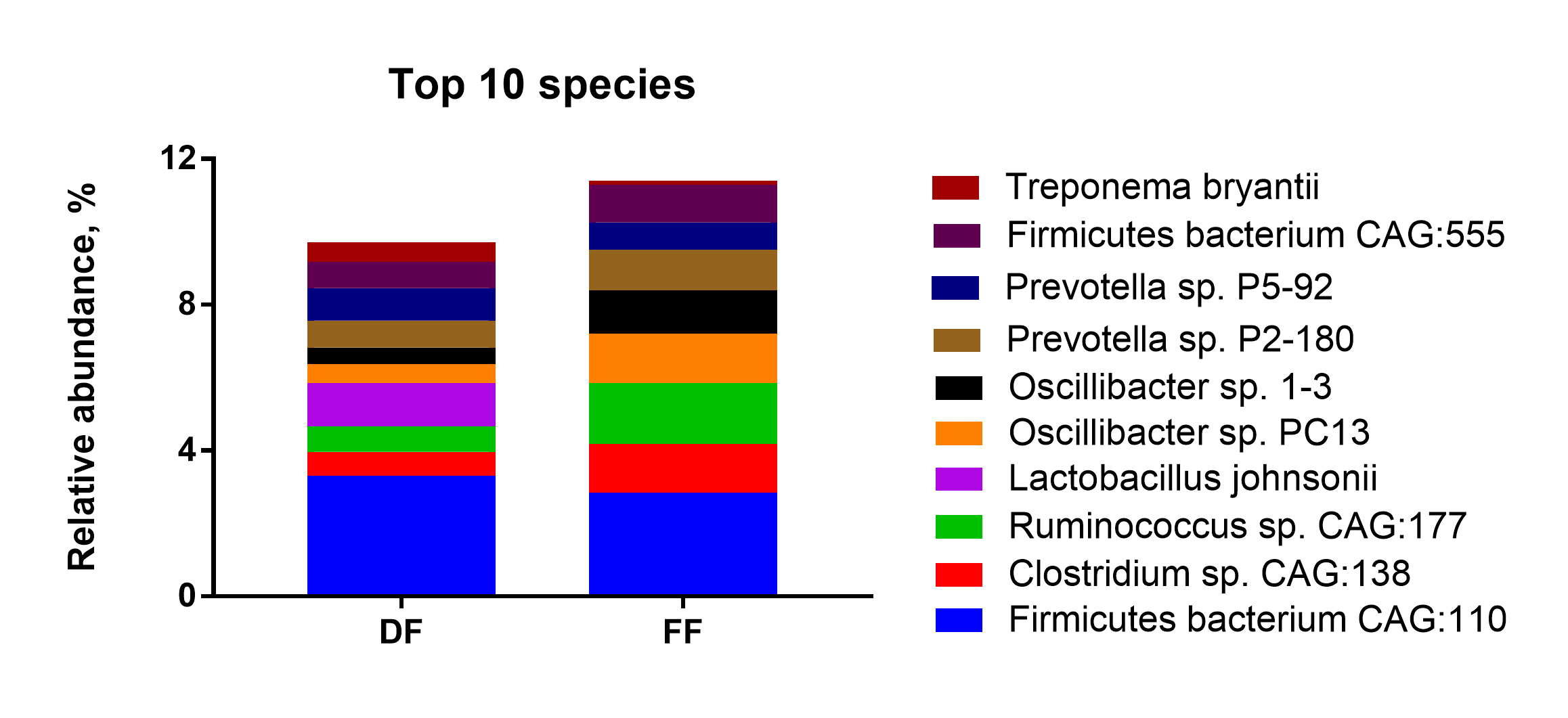

Supplement: Supplementary file 2 — Figure S2. Relative abundances of the bacteria in the top 10 species level taxa of the DF and FF groups. (TIF 275 kb) [file 12866_2019_1556_MOESM2_ESM.tif]

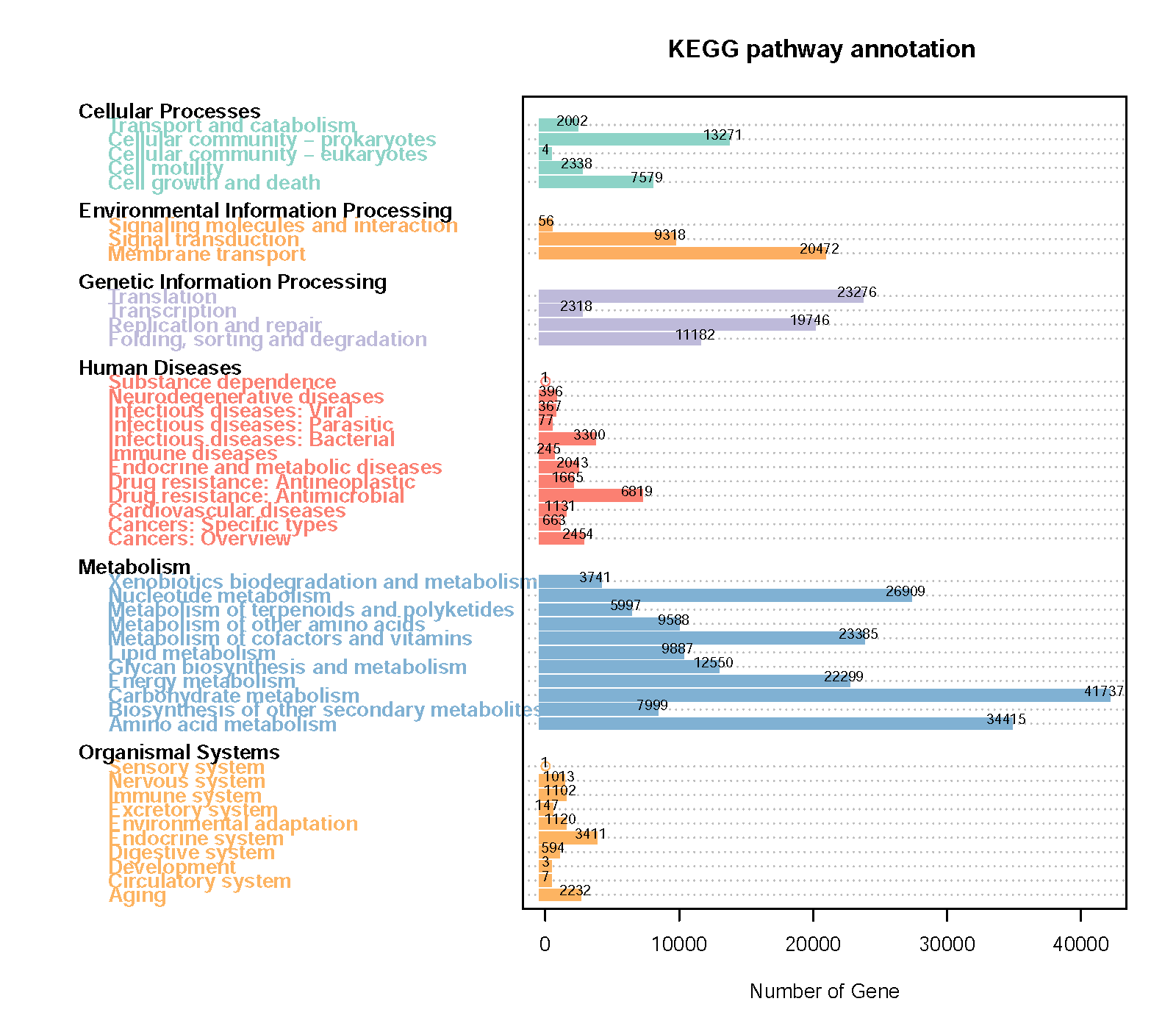

Supplement: Supplementary file 3 — Figure S3. Functional classification of annotated NR genes by using KEGG analysis. (TIF 304 kb) [file 12866_2019_1556_MOESM3_ESM.tif]
