# Supplementary material for: Rearing pattern alters porcine myofiber type, fat deposition, associated microbial communities and functional capacity
Source: BMC Microbiol. 2019 Aug 6;19:181. doi: 10.1186/s12866-019-1556-x (PMC6683424; doi:10.1186/s12866-019-1556-x)
Supplement: Supplementary file 6 — Table S3. Composition of the basal diets in the traditional feeding farm. Table S4. Composition of the basal diets the semi free-grazing farm. Table S5. Primer sequences (5′to 3′) used for the quantitative polymerase chain reaction. (DOC 59 kb) [file 12866_2019_1556_MOESM6_ESM.doc]

Supplementary tables:

Table S3. Composition of the basal diets in the traditional feeding farm (%)

| Ingredients | <30kg | 30~60kg | >60kg |
| --- | --- | --- | --- |
| Maize | 52 | 67 | 50 |
| Soybean meal | 27 | 21 | 18 |
| Barley | 12 | 0 | 20 |
| Soybean oil | 3 | 0 | 0 |
| Fish meal | 2 | 0 | 0 |
| Rapeseed meal | 0 | 4 | 4 |
| Rice bran | 0 | 4 | 4 |
| Dicalcium phosphate | 0.65 | 0.60 | 0.2 |
| Calcium carbonate | 1 | 1.2 | 1.2 |
| Salt | 0.4 | 0.5 | 0.56 |
| Choline chloride | 0.1 | 0.1 | 0 |
| Lysine | 0.2 | 0.16 | 0.13 |
| Methionine | 0.1 | 0.02 | 0.01 |
| Threonine | 0.02 | 0 | 0 |
| Trace mineral premix^1^ | 0.53 | 0.42 | 0.9 |
| Vitamin premix^2^ | 1 | 1 | 1 |

^1^Providing per kg of diet for body weight <30 kg: Cu (as CuSO_4_·5H_2_O) 125 mg, Zn (as ZnSO_4_·7H_2_O) 100 mg, Mn (as MnSO_4_·H_2_O) 50 mg, Se (as Na_2_SeO_3_) 0.3 mg, I (as Ca(IO_3_)_2_) 0.4 mg; diet for body weight of 30–60 kg: Cu (as CuSO_4_·5H_2_O) 75 mg, Zn (as ZnSO_4_·7H_2_O) 80 mg, Mn (as MnSO_4_·H_2_O) 35 mg, Se (as Na_2_SeO_3_) 0.3 mg, I (as Ca(IO_3_)_2_) 0.4 mg; diet for body weigh >60 kg: Cu (as CuSO_4_·5H_2_O) 25 mg, Zn (as ZnSO_4_·7H_2_O) 70 mg, Mn (as MnSO_4_·H_2_O) 30 mg, Se (as Na_2_SeO_3_) 0.3 mg, and I (as Ca(IO_3_)_2_) 0.4 mg.

^2^Providing per kg of diet for body weight <30 kg: retinol acetate 7500 IU, cholecalciferol 750 IU, DL-α-tocopherol acetate 25 IU, menadione 2 mg, thiamine 1.875 mg, riboflavin 3.75 mg, pyridoxine 2.19 mg, cobalamin 0.025 mg, pantothenic acid 15.6 mg, niacin, 25 mg, folic acid 2.0 mg, biotin 0.1875 mg; diet for body weight of 30–60 kg: retinol acetate 6000 IU, cholecalciferol 600 IU, DL-α-tocopherol acetate 20 IU, menadione 1.6 mg, thiamine 1.5 mg, riboflavin 3 mg, pyridoxine 1.75 mg, cobalamin 0.02 mg, pantothenic acid 12.5 mg, niacin, 20 mg, folic acid 1.6 mg, biotin 0.15 mg; diet for body weigh >60 kg: retinol acetate 5250 IU, cholecalciferol 525 IU, DL-α-tocopherol acetate 17.5 IU, menadione 1.4 mg, thiamine 1.3 mg, riboflavin 2.6 mg, pyridoxine 1.5 mg, cobalamin 0.0175 mg, pantothenic acid 10.9 mg, niacin, 17.5 mg, folic acid 1.4 mg, and biotin 0.13 mg.

Table S4. Composition of the basal diets the semi free-grazing farm (%)

| Ingredients | Percentage |
| --- | --- |
| Maize | 65 |
| Soybean meal | 12 |
| Rice Bran | 10 |
| Broken rice | 5 |
| Wheat bran | 7 |
| Dicalcium phosphate | 1 |
| Supplementary feeding: seasonal weeds, pastures or vegetables | |

Table S5. Primer sequences (5′ to 3′) used for the quantitative polymerase chain reaction

| Gene | Forward pride | Reversed prime |
| --- | --- | --- |
| FAS | CCTGTATCGCTGGACCACT | GGGCACTCAGACTCCCTTT |
| ATGL | ACCTTCATTCCCGTGTACTGCG | GGTGATGGTGCTCTTGAGTTCGT |
| HSL | TCTGGAATATCACCGAGATTGAG | CATAGGAGATGAGCCTGACGAG |
| A-FABP | TGAAAGGTGTCACGGCTAC | TCGGGACAATACATCCAACAG |
| SCD | TCTGGGCGTTTGCCTACTATCT | TCTTTGACGGCTGGGTGTTT |
| ADPR | TGTGGATGCGGAAGATGC | CCACCAACCCACCCAAAG |
| SOCS3 | CGACACCTCTTCACGCTCAG | TGGCATGTAGTGACGCACC |
| CAST | ATCGCCTTCCTCTGCTTC | TTGGCTCTGGGTGTTCCT |
| UCP3 | TCGGACCACTCCAGCATCA | CCATCTCGGCACAGTTCACG |
| MyHC1 | CACTTGCTAAGAGGGACCTCTGAGTTCA | ATCCAGGCTGCGTAACGCTCTTTGAGGTTGTA |
| MyHC2a | AGCCTCTTTCTTCTCCCAGGGACATTC | ATCCAGGCTGCGTAACGCTCTTTGAGGTTGTA |
| MyHC2b | CATCTGGTAACATAAGAGGTACATCTAG | ATCCAGGCTGCGTAACGCTCTTTGAGGTTGTA |
| MyHC2x | CTTTCCTCATAAAGCTTCAAGTTCTGCC | ATCCAGGCTGCGTAACGCTCTTTGAGGTTGTA |
| FOXO1 | TACGCCGACCTCATCACCA | TCCCACTCTTGCCTCCCTCT |
| MyoD1 | TTCTATGATGACCCGTGTTTCG | CGTTAGTGGTCTTGCGTTTGC |
| GADPH | TCTGGCAAAGTGGACATT | GGTGGAATCATACTGGAACA |
